# A long-term observational study of paediatric snakebite in Kilifi County, south-east Kenya

**DOI:** 10.1371/journal.pntd.0010987

**Published:** 2023-07-17

**Authors:** Michael Abouyannis, Mwanamvua Boga, David Amadi, Nelson Ouma, Amek Nyaguara, Neema Mturi, James A. Berkley, Ifedayo M. Adetifa, Nicholas R. Casewell, David G. Lalloo, Mainga Hamaluba

**Affiliations:** 1 KEMRI-Wellcome Trust Research Programme, Centre for Geographic Medicine Research (Coast), Kilifi, Kenya; 2 Centre for Snakebite Research and Interventions, Liverpool School of Tropical Medicine, Liverpool, United Kingdom; 3 Centre for Tropical Medicine & Global Health, Nuffield Department of Medicine, Oxford, United Kingdom; 4 London School of Hygiene and Tropical Medicine, London, United Kingdom; Fundação de Medicina Tropical Doutor Heitor Vieira Dourado, BRAZIL

## Abstract

**Introduction:**

Estimates suggest that one-third of snakebite cases in sub-Saharan Africa affect children. Despite children being at a greater risk of disability and death, there are limited published data. This study has determined the: population-incidence and mortality rate of hospital-attended paediatric snakebite; clinical syndromes of snakebite envenoming; and predictors of severe local tissue damage.

**Methods:**

All children presenting to Kilifi County Hospital, Kenya with snakebite were identified through the Kilifi Health and Demographic Surveillance System (KHDSS). Cases were prospectively registered, admitted for at least 24-hours, and managed on a paediatric high dependency unit (HDU). Households within the KHDSS study area have been included in 4-monthly surveillance and verbal autopsy, enabling calculation of population-incidence and mortality. Predictors of severe local tissue damage were identified using a multivariate logistic regression analysis.

**Results:**

Between 2003 and 2021, there were 19,606 admissions to the paediatric HDU, of which 584 were due to snakebite. Amongst young children (≤5-years age) the population-incidence of hospital-attended snakebite was 11.3/100,000 person-years; for children aged 6–12 years this was 29.1/100,000 person-years. Incidence remained consistent over the study period despite the population size increasing (98,967 person-years in 2006; and 153,453 person-years in 2021). Most cases had local envenoming alone, but there were five snakebite associated deaths. Low haemoglobin; raised white blood cell count; low serum sodium; high systolic blood pressure; and an upper limb bite-site were independently associated with the development of severe local tissue damage.

**Conclusion:**

There is a substantial burden of disease due to paediatric snakebite, and the annual number of cases has increased in-line with population growth. The mortality rate was low, which may reflect the species causing snakebite in this region. The identification of independent predictors of severe local tissue damage can help to inform future research to better understand the pathophysiology of this important complication.

## Author summary

The nature of disease caused by snakebite amongst children in rural Africa is poorly understood. All children presenting to Kilifi County Hospital, Kenya with snakebite between 2003 and 2021 were recruited into a study that was nested in an ongoing hospital and community surveillance study. 584 children presented following snakebite and the incidence was 29.1/100,000 person-years amongst children aged 6–12 years. The incidence remained consistent over the course of the study despite the population increasing by over 50%, highlighting that the burden of disease is increasing in-line with population growth. Many children initially sought traditional therapies, which was associated with delayed presentation to hospital. The mortality rate was low, and most children had local, rather than systemic, envenoming. Predictors of severe local envenoming at the time of admission included a high white blood cell count, low haemoglobin, and an upper limb bite site. The findings of this study highlight the need to strengthen strategies to prevent snakebite in children, to overcome barriers to attending hospital, and to develop therapeutics that better treat local envenoming.

## Introduction

Snakebite is a neglected tropical disease that affects 5 million people each year, with the greatest burden falling on rural populations of the tropics and sub-tropics [1]. Snakebite disproportionately affects children living in low-income countries, who are likely to be at a greater risk of disability and mortality. In sub-Saharan Africa it has been estimated that 30% of people affected by snakebite are children [2]. Typical activities that may bring children into contact with snakes include outdoor play, agricultural work, and walking to school. The burden of snakebite in sub-Saharan Africa has been estimated at over 1 million DALYs (disability-adjusted life years) per year [3]. Much of this is accounted for by deaths and limb amputations in the young, who disproportionately contribute to disability adjusted life years and years of life lost [3].

The limited available evidence suggests that children are twice as likely to be administered antivenom following snakebite and have an increased risk of death [4–6]. It is hypothesised that children are particularly vulnerable as they receive a higher dose of venom relative to their body weight. Despite this, limited studies have described the burden of paediatric snakebite in sub-Saharan Africa. Two observational studies in South Africa (with samples sizes of 51 and 72 children) identified a substantial burden of painful progressive swelling, with one in four cases undergoing debridement or fasciotomy [7,8]. In a retrospective study of 28 consecutive cases of paediatric snakebite in The Gambia, the mortality rate was 14% [9]. In a community based survey conducted in Kilifi County, Kenya, in 1994, 46% of snakebite cases occurred in children or young adults [10].

Kilifi County is situated on the south-east coast of Kenya and is the site of the KEMRI (Kenya Medical Research Institute)-Wellcome Trust Research Programme. This programme conducts medical research and is formed of a partnership between KEMRI, the University of Oxford, and the Wellcome Trust. The predominant biting snake species have not been systematically defined in Kilifi County, although several medically important species are present in this region, including the puff adder (*Bitis arietans)*, mambas (*Dendroaspis polylepis*, *D*. *angusticeps*) and spitting cobras (*Naja ashei*, *N*. *pallida*). Through a partnership between the Kilifi County Department of Health and the KEMRI-Wellcome Trust Research Programme, the Kilifi Health and Demographic Surveillance System (KHDSS) has prospectively recorded data, and post-discharge outcomes, on paediatric admissions (including snakebite cases) to Kilifi County Hospital since 2003 [11]. In this study, comprehensive data have been gathered, from consecutive snakebite cases between 2003 and 2021, to describe the annual population-incidence of hospital-attended paediatric snakebite in Kilifi County, calculate the mortality rate, describe the clinical syndromes of envenoming, and to identify independent predictors of severe local tissue damage.

## Methods

### Ethical approvals

Written informed consent, with verbal and written patient information translated to Swahili and Giriama, was obtained from the parent or guardian of children enrolling in to the Kilifi Health and Demographic Surveillance System (KHDSS) hospital surveillance study. The KHDSS study has approval from the Kenya Medical Research Institute Scientific Ethics Review Unit [KEMRI SERU] (KEMRI/SERU/CGMR-C/3057), which was most recently renewed on the 26th of September 2022. Approval to undertake this study, including the extraction of data from the hospital records, was granted by the Kenya Medical Research Institute Scientific Ethics Review Unit on the 4^th^ of November 2019 [KEMRI SERU] (KEMRI/SERU/CGMR-C/174/3930) and the Liverpool School of Tropical Medicine Research Ethics Committee [LSTM REC] (19–064) on the 21^st^ of November 2019.

### Study site

The KHDSS hospital surveillance system has collected data on paediatric admissions (including snakebite cases) to Kilifi County Hospital since 2003. Paediatric care in Kenya is provided to children aged ≤12-years. To avoid misclassifying cases with delayed onset of clinical envenoming, local policy stipulates that all cases of paediatric snakebite are admitted for ≥24-hours observation on the paediatric high dependency unit (HDU). This is regardless of disease severity and even applies to cases with no features of envenoming. The paediatric HDU is funded and staffed by the KEMRI-Wellcome Trust Research Programme and a standardised protocol for the management of snakebite is in place. The resources and quality of care available at this paediatric HDU are higher than typical government healthcare facilities in much of Africa, facilitating an approach to snakebite management that aligns with recommended standards in high-income settings. Antivenom is administered in accordance with WHO guidelines [12]. Once paediatric cases with snakebite have been managed on the paediatric HDU for 24-hours, the clinical team decide whether it is appropriate for the child to remain on the paediatric HDU, be stepped down to the Kilifi County Hospital paediatric ward, or to be discharged home. Cases transferred to the paediatric ward continue to have data collected as part of the KHDSS hospital surveillance study, and cases discharged home are followed-up by the KHDSS community surveillance system.

Hospital surveillance data are linked to the KHDSS community surveillance data. The study area is 891 km^2^ and Kilifi County Hospital is the only hospital with inpatient paediatric services in the study area [11]. The KHDSS study area was defined based on the lowest number of administrative sublocations that were the site of residence of greater than 80% of the paediatric inpatients at Kilifi County Hospital over a three-year period (1998–2000) [11]. The study area, including all dwellings, has been GPS mapped. In 2021, the KHDSS included 92,063 households and 309,228 residents. Most of the study population reside in rural dwellings and the local economy is predominantly centred on subsistence farming [11].

### Identification and eligibility of cases

All cases of snakebite affecting children aged ≤12 years, attending from January 2003 until December 2021, were eligible for inclusion in this study. These cases were routinely enrolled into the KHDSS hospital surveillance study over this period. At admission and on discharge from Kilifi County Hospital, clinical-research staff prospectively assigned a diagnostic code which was recorded on the KHDSS hospital database. Specific diagnostic codes were in place to classify snakebite, as follows: ‘snake venom’ and ‘snake bite.’ As a precaution, to avoid missing cases that may have been incorrectly classified, database search terms also included the following diagnostic codes, which were recorded at admission and on discharge: (1) “snake venom”; (2) “snake bite”; or (3) “acute animal bite.” In addition to searching diagnostic codes, free-text sections of the database were searched for the following terms: (1) “snake”; or (2) “venom”. All cases identified through this database search were screened by an academic clinician (MA) and non-snakebite cases, for example a dog bites, were excluded. For a case to be included there had to be a specific and clear reference to snakebite being the cause of the admission, in the database or the clinical records. In cases where the diagnosis of snakebite was uncertain, the paper notes were scrutinised by the study team.

### Data extraction

The following data were prospectively recorded on the KHDSS database at the time of hospital admission by research staff: demographics; date and time of admission; date and time of discharge; weight; admission vital signs; diagnosis code; mortality; and date of death. The following clinical laboratory results from admission samples were extracted from the KEMRI-Wellcome Trust Research Programme laboratory database: full blood count (including differential); serum sodium; serum potassium; and serum creatinine.

To supplement the above prospective data that were collected in the KHDSS, retrospective data from the paper case notes were extracted by a team of research nurses using a standardised case report form. The following retrospective data were extracted: residence; geographic location of bite; circumstances of bite; date and time of bite; anatomical location of bite; use of traditional treatments; clinical features of local and systemic envenoming; clotting time; antivenom administration; indication for antivenom; antivenom associated adverse events; adjunctive treatments; complications of envenoming; and discharge destination. The antivenom product that was administered was not documented in the medical records. However, from the hospital pharmacy records it was possible to identify the antivenom product available during each study year.

Deaths were identified by searching the KHDSS hospital database, the paper medical records, and the KHDSS community database. Verbal autopsy was routinely conducted for all deaths that occurred within the KHDSS study area. This was conducted using the 2007 World Health Organization (WHO) verbal autopsy tools [13], as described previously [14].

To calculate the population-incidence of hospital-attended snakebite, census data from the KHDSS community database were used. Full details of this surveillance system have previously been published [11]. Community interviewers visited every household in the study area on a 4-monthly basis. A single resident was interviewed, from whom information pertaining to each resident was collected. The identity of all residents was confirmed and any newly born children, in-migrations, deaths, and out-migrations, since the previous enumeration round, were recorded. Person-years of observation were stratified by sex, age and 41 geographic sub-locations.

### Statistical analysis

Clinical data were described using summary statistics including means, medians, and proportions. Population-incidence of hospital-attended snakebite was calculated with 95% confidence intervals. Population incidence was calculated separately for young children (0–5 years inclusive) and older children (6–12 years inclusive). The hospital surveillance system did not consistently capture all admissions until 2006, thus, to avoid underestimation, incidence estimates have only been calculated for the period of 2006–2021.

A logistic regression analysis was conducted to identify variables associated with severe local tissue damage. Severe local tissue damage was defined as any case developing local skin necrosis or requiring surgical intervention–criteria that are congruent with the recently established snakebite core outcome measurement set [15]. The following variables were included in a univariate analysis, and those with a significance value of p ≤0.10 were selected for inclusion in the multivariate analysis: age, site of bite (dichotomised as upper limb and lower limb), elapsed time from bite to admission, MUAC (mid-upper arm circumference, which was measured away from the bite site)-for-age z-score (using the *zscorer* package in R), vital signs on admission (pulse rate, respiratory rate, systolic blood pressure, capillary refill time, axillary temperature, and oxygen saturations), admission full blood count (haemoglobin, white cell count, granulocyte count, lymphocyte count, and platelet count), and admission serum biochemistry (sodium, potassium, and estimated glomerular filtration rate [eGFR]). The eGFR was calculated using the Schwartz equation [16]. Multiple imputation, using the *mice* package in R, was undertaken to replace missing values [17]. R version 4.2.2 (R Foundation for Statistical Computing) was used for all analyses.

## Results

### Incidence of hospital-attended paediatric snakebite

**Fig 1:**
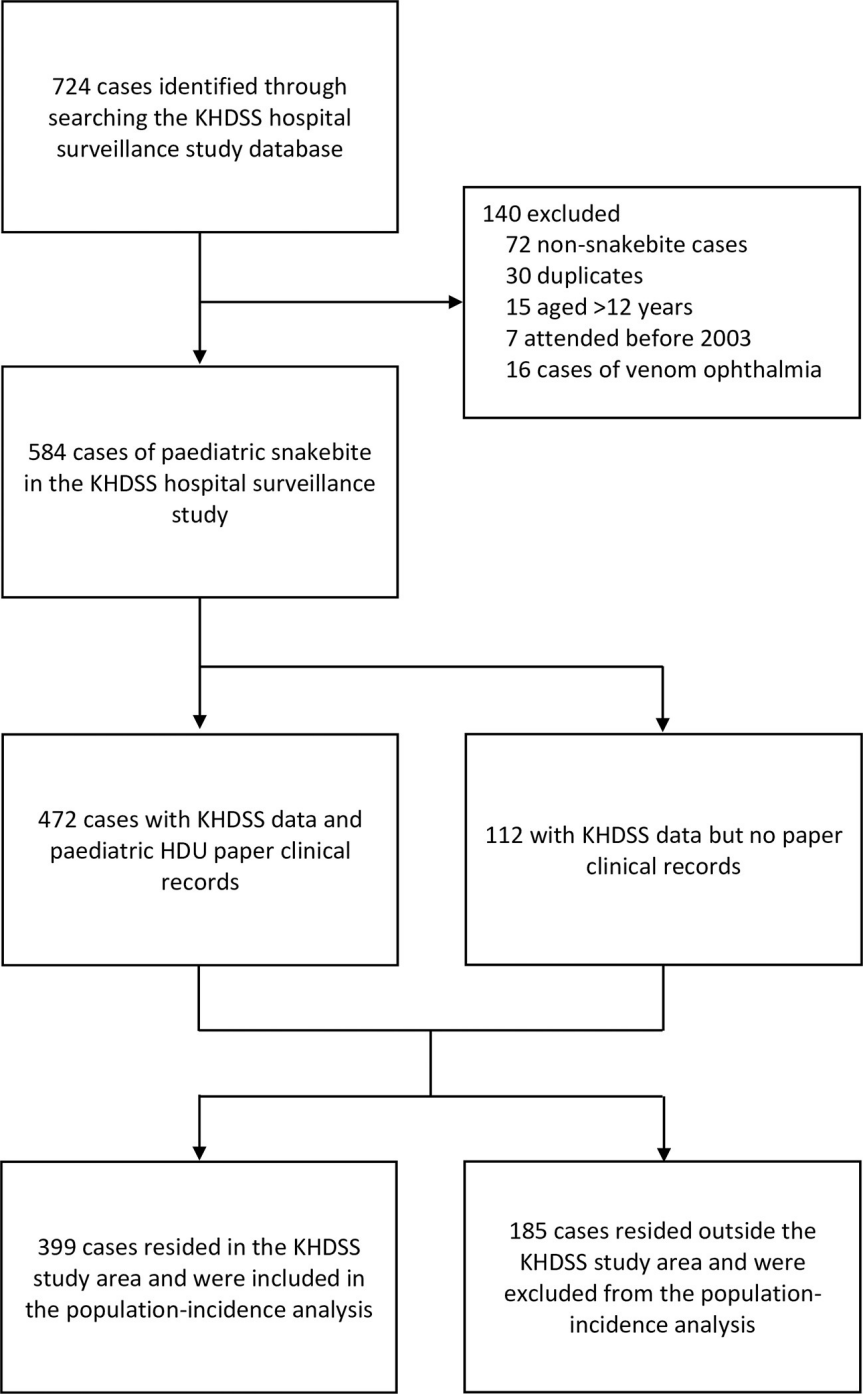
CONSORT diagram of participant inclusions and exclusions. KHDSS: Kilifi Health and Demographic Surveillance System.

During the study period there were 78,038 paediatric admissions to Kilifi County Hospital (children aged ≤12-years), and 19,606 admissions to the paediatric HDU. The diagnostic code search of the KHDSS hospital database identified 724 potential paediatric snakebite cases. Following manual review of clinical data, 72 were excluded as they were not cases of snakebite (these were predominantly bites by other animals). Further exclusions are detailed in the CONSORT diagram (Fig 1). There were 584 children aged 12-years or under that presented with snakebite to Kilifi County Hospital between January 2003 and December 2021. Snakebite thus represented 2.98% of all admissions to the paediatric HDU over the study period. Details of the proportion of admissions to Kilifi County Hospital for snakebite by year are available in Table A in S1 Text. The median age was 8 years (IQR 5–10 years) and 47.6% were female. Clinical records were available for 472 (80.8%) participants, and most were resident in the KHDSS study area (N = 399; 68.3%).

**Fig 2:**
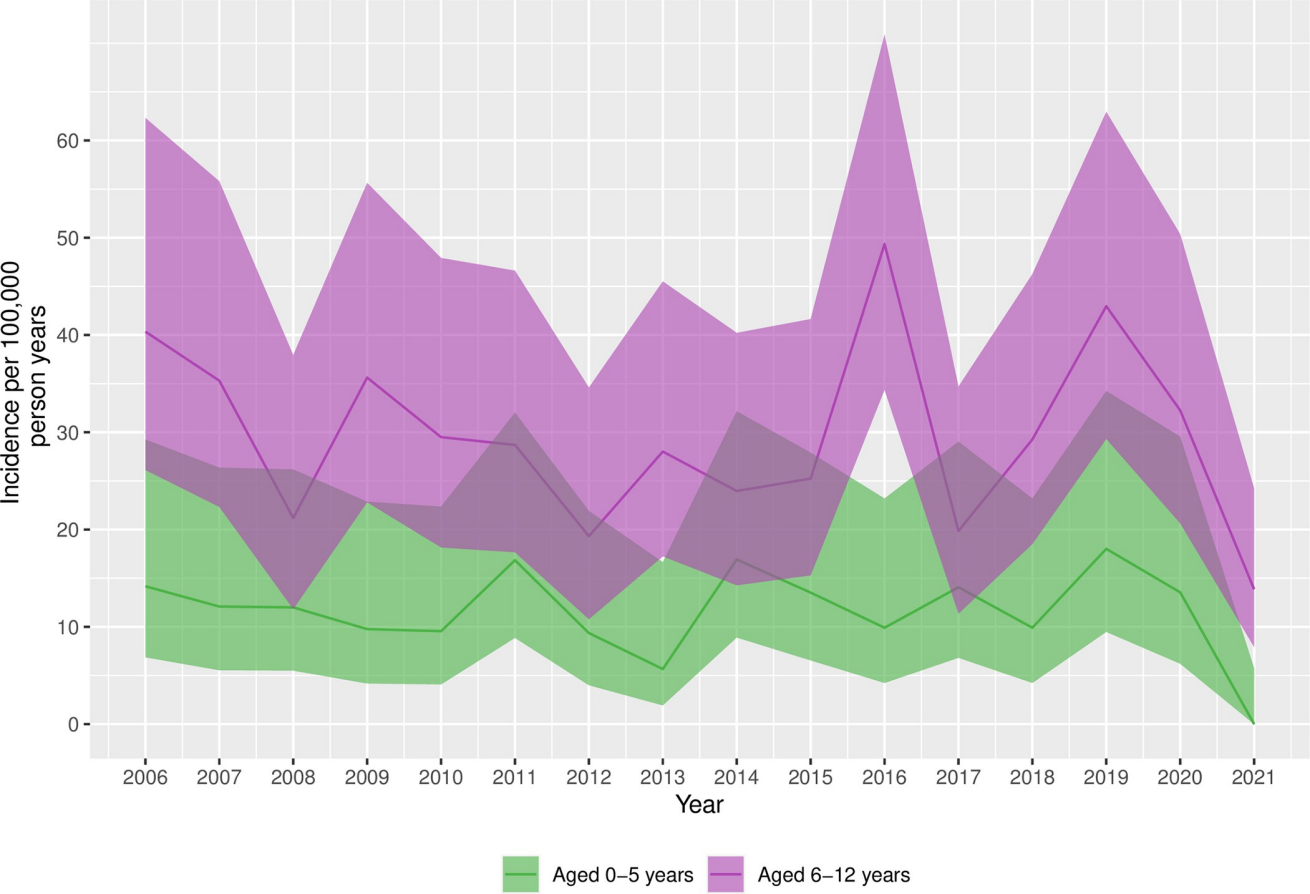
Annual population-incidence of hospital-attended paediatric snakebite. Population incidence is presented per 100,000 person-years of follow-up. The numerator is represented by the number of children presenting to Kilifi County Hospital each year with snakebite. The denominator is the number of person-years of follow-up recorded in the Kilifi Health and Demographic Surveillance System study area, which is recorded through household survey every 4-months. The bands represent the 95% confidence intervals. Population incidence is stratified by age category.

The population-incidence of hospital-attended snakebite was calculated using the number of admissions per year amongst children that resided in the KHDSS study area (numerator) and annual age-specific census data from the KHDSS (denominator). As young children had a substantially lower risk of snakebite, population-incidence was stratified between the ages 0-5-years and 6-12-years. For children aged ≤5-years, the average population-incidence between 2006 and 2021 was 11.3/100,000 person-years; for children aged 6-12-years, the average population-incidence was 29.1/100,000 person-years. Fig 2 demonstrates the annual population-incidence for young children and older children, with 95% confidence intervals. Although there is variability between study years, the incidence remained broadly consistent until 2020, with a decline in 2021. As there has been a substantial increase in the number of people residing in the KHDSS study area, absolute numbers presenting with snakebite have increased over the study period. In 2006 there were 98,967 person-years of follow-up amongst children ≤12 years of age; by 2021 this had increased to 153,453 person-years.

**Fig 3:**
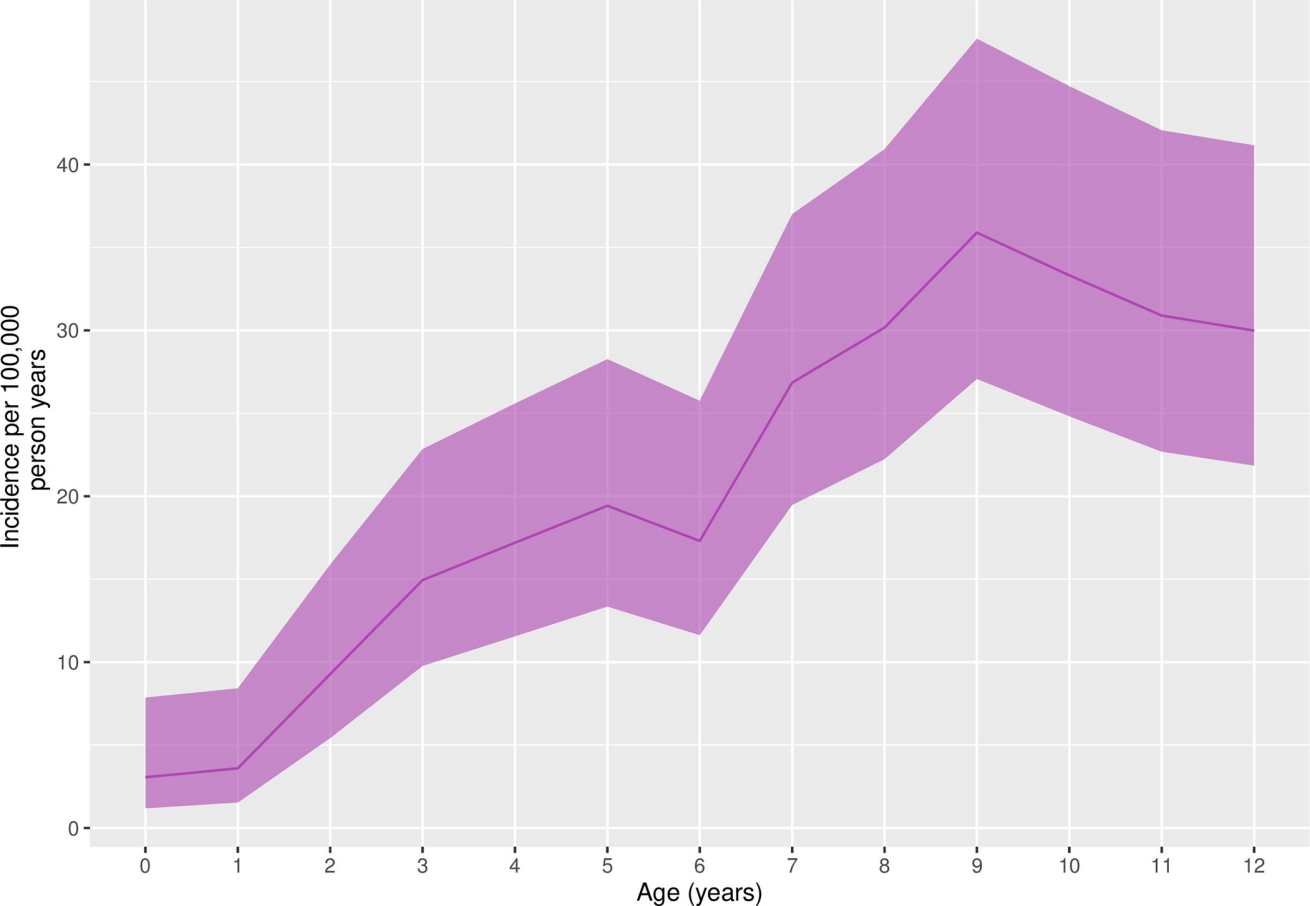
Population incidence of hospital-attended paediatric snakebite by age. Population incidence is presented per 100,000 person-years of follow-up. The numerator is represented by the average annual number of children presenting to Kilifi County Hospital with snakebite between 2006 and 2021, stratified by year of age. The denominator is the average annual number of person-years of follow-up recorded in the Kilifi Health and Demographic Surveillance System study area between 2006 and 2021, stratified by year of age. The band represents the 95% confidence interval.

Population-incidence of hospital-attended snakebite was calculated by year of age, as shown in Fig 3. There was a substantial increase in incidence with age: from 3.6/100,000 person-years at age 1-year, to 35.9/100,000 person-years at age 9-years. With increasing age above 9-years incidence fell, reaching 30.0/100,000 person-years by age 12-years.

### Clinical features

**Fig 4:**
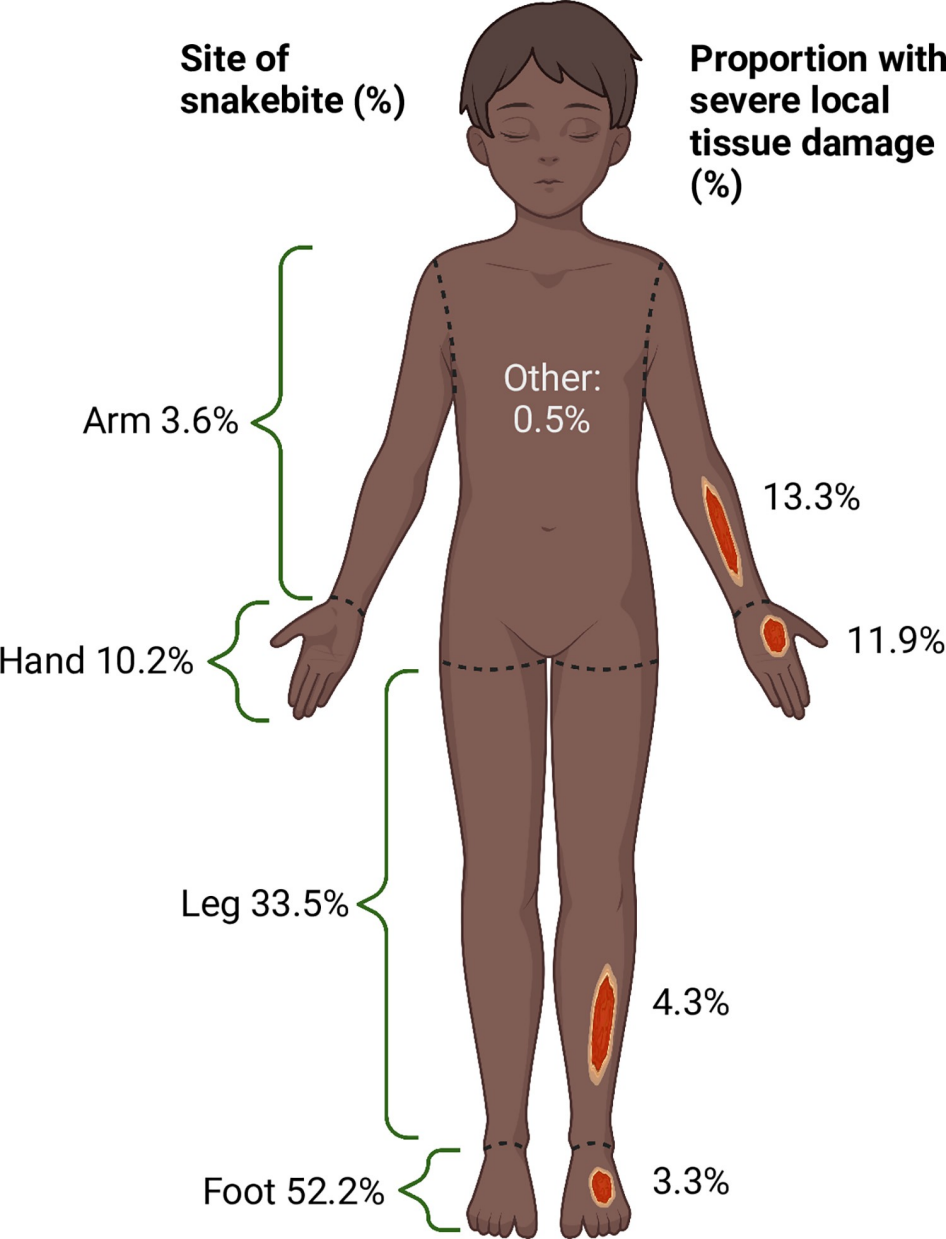
Overview of the anatomical locations of paediatric snakebite in Kilifi County and the proportion developing severe local tissue damage at each site. The left side of the image shows the proportion of children bitten at each anatomical site. The central 0.5% section represents the two children with non-limb bites. The right side of the image describes the proportion of bites at each anatomical site that were associated with severe local tissue damage (defined as developing skin necrosis or requiring local surgery). Created with BioRender.

The circumstances of the snakebite were available in the clinical records in 307 (52.6%) cases. Most snakebites occurred outdoors and near to the child’s home (131 cases; 42.7%) or in the child’s house (70 cases; 22.8%). Amongst cases with a documented site of injury, the majority involved the feet or legs (Fig 4).

Traditional therapies that were sought prior to admission included application of a ‘black stone’ in 110 (23.3%) cases, application of a torniquet in 48 (10.2%) cases, and cutting the skin at the bite site in 47 (10.0%) cases (Table 1). Two or more types of traditional therapy were sought prior to admission in 56 (11.9%) cases (S1 Fig). The median elapsed time from bite until admission was 6-hours and 45-minutes (IQR 3-15-hours; range 10 minutes-17 days). The median elapsed time from admission until antivenom administration was 2-hours and 50-minutes (IQR 1–9 hours). Children who had received traditional therapies took a median of 3.6 hours longer to present to hospital, and this difference was statistically significant (median 9.4 hours and 5.8 hours; p = 0.003).

**Table 1:** Characteristics of paediatric snakebite cases presenting to Kilifi County Hospital.

**Hospital surveillance data**	**N = 584**
Median age (IQR), years	8 (5–10)
<1 year, n (%)	7 (1.2)
1 year, n (%)	12 (2.1)
2–5 years, n (%)	139 (23.8)
6–9 years, n (%)	238 (40.8)
10–12 years, n (%)	188 (32.2)
Sex, n (%), females	278 (47.6)
Abnormal age specific vital signs, n (%)	
Tachycardia (age adjusted)	311 (53.3)
Tachypnoea (age adjusted)	345 (59.3)
Hypotension (age adjusted)	21 (4.6)
Capillary refill >2 seconds	9 (1.6)
Hypoxia (saturations <95%)	11 (1.9)
Fever >37.2°C	158 (27.1)
Severe local tissue damage, n (%)	25 (4.3)
Local necrosis	22 (3.8)
Required surgery	19 (3.3)
Debridement	10 (1.7)
Fasciotomy	7 (1.2)
Skin graft	4 (0.7)
Amputation	3 (0.5)
Snakebite associated mortality, n (%)	5 (0.9)
**Clinical record data, n (%)**	**n = 472 (80.8)**
Circumstances of bite, n (%)	
Outside home compound	131 (42.7)
Inside home	70 (22.8)
Walking on footpath	51 (16.6)
Farming	30 (9.8)
Walking in bushland	14 (4.6)
At school	6 (2.0)
Hunting for small mammals in burrows	5 (1.6)
Missing	165
Traditional therapies, n (%)	
Black stone	110 (23.3)
Torniquet	48 (10.2)
Puncture to bite site	47 (10.0)
Herbal medicine	42 (8.9)
Suction	3 (0.6)
Clinical features, n (%)	
Swelling	399 (84.5)
Blisters	40 (8.5)
Bite site bleeding	18 (3.8)
Bite site infection	9 (1.9)
Systemic bleeding	6 (1.3)
Neurotoxicity	2 (0.4)
No features of envenoming	51 (10.8)

IQR: inter-quartile range; KHDSS: Kilifi Health and Demographic Surveillance System.

Most children had local swelling at presentation, being present in 399 (84.5%) cases (Table 1). There were six cases with systemic bleeding, and two with neurotoxic envenoming. There were no features of envenoming in 51 cases (10.8%). Age-adjusted tachycardia, hypotension, and tachypnoea were present in 311 (53.3%), 21 (4.6%), and 345 (59.3%) cases, respectively. The 20-minute whole blood clotting test (20-WBCT) was documented in only 18 cases. Many were conducted incorrectly and followed a procedure akin to the Lee-White clotting time (with repeated checks of the sample before 20-minutes had elapsed). Two of the 18 cases where a bedside clotting test was documented were prolonged over 20-minutes.

**Fig 5:**
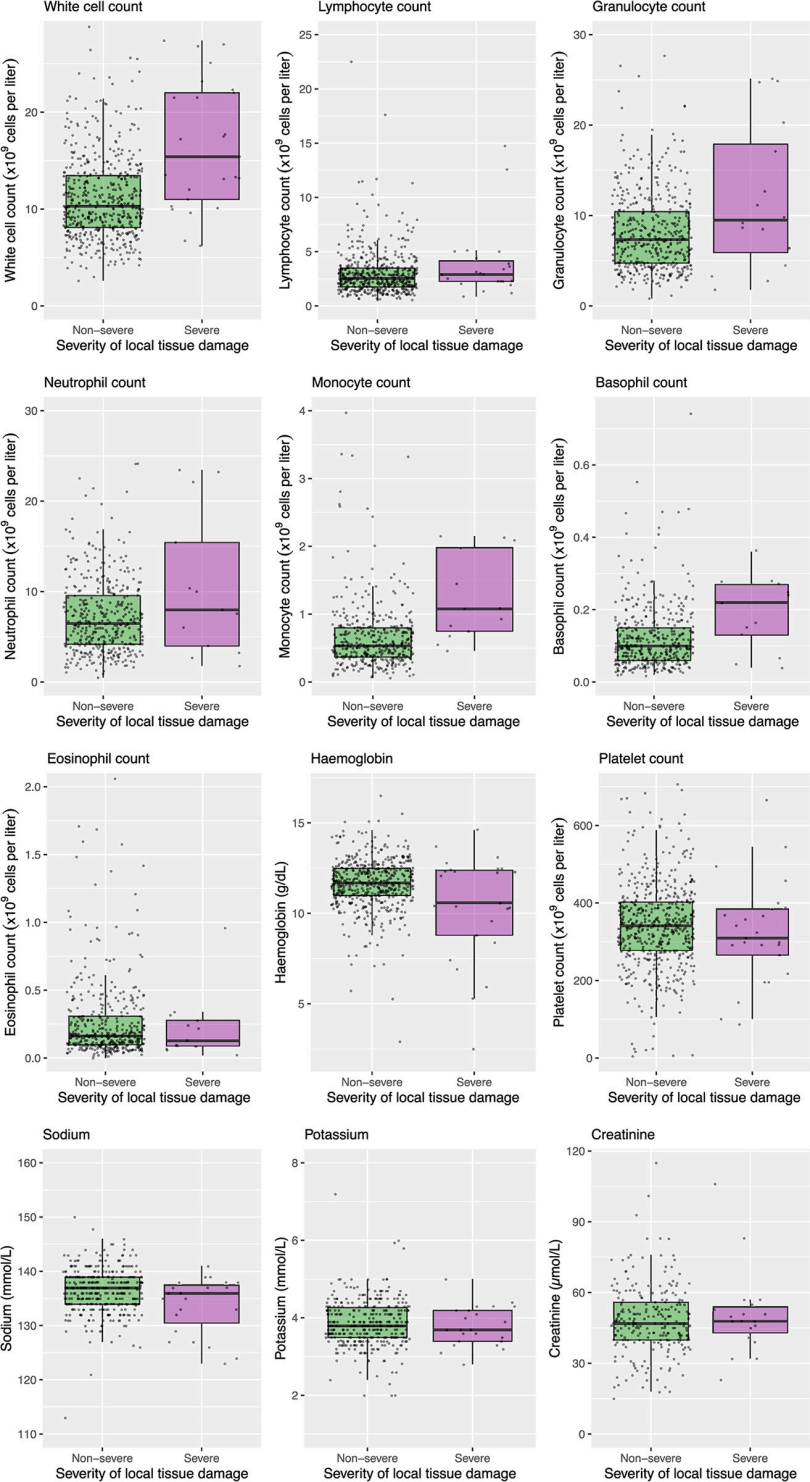
Boxplots of clinical laboratory values on admission to hospital stratified by development of snakebite associated severe local tissue damage. Boxplots represent the distribution of blood results stratified by participants with and without severe local tissue damage (defined as developing skin necrosis or requiring local surgery). The box details the median, the 25th percentile, and the 75th percentile. The whiskers extend to the highest value that is within 1.5x the interquartile range from the edge of the box. All data points are overlayed and scattered horizontally to aid visualisation.

Full blood count and serum biochemistry were routinely undertaken for all children presenting with snakebite. In cases where insufficient blood sample volumes were obtained, due to challenging access, the full blood count was performed in preference to biochemistry. These demonstrated anaemia (haemoglobin <8.2 g/dL) in 51 (9.6%) cases, leukocytosis in 314 (59.5%) cases, and reduced eGFR in 12 (5.0%) cases (Table B in S1 Text). The clinical laboratory results, stratified by severe local tissue damage, have been depicted in Fig 5. The numbers of children with age-adjusted abnormal clinical laboratory results have been summarised in Table B in S1 Text.

### Hospital management

Antivenom was administered to 119 (25.2%) children with snakebite (Table 2). Stratified by age, antivenom was administered to 43 of 127 (33.9%) children aged ≤5-years, and 76 of 345 (22.0%) children aged 6-12-years. A single vial was administered to 72 (67.9%) children, 26 (24.5%) children received two vials, and 8 (7.5%) children received three or more vials. Fav-Afrique (Sanofi Pasteur) was administered to 73 children, 12 received ASNA (Bharat Serums and Vaccines), 9 received Snake Venom Antiserum African (VINS Bioproducts), and 25 received Inoserp Pan-Africa (Inosan Biopharma) polyvalent antivenoms (Table 2).

**Table 2:** Antivenom products administered, numbers of vials, and rates of acute allergic reactions.

Date range	Available antivenom product	Admitted, n	Received antivenom, n (%)	Number of vials administered, n (%)	Number with adverse reactions, n (%)
2003–2014	Fav-Afrique (Sanofi Pasteur) polyvalent antivenom	287	73 (25.4%)	1 vial: 52 (77.6%) 2 vials: 12 (17.9%) ≥3 vials: 3 (4.5%)	Any reaction: 13 (17.8%) Urticarial rash: 12 (16.4%) Fever: 1 (1.4%) Bronchospasm: 1 (1.4%) Hypotension: 0 (0%)
2015–2016	ASNA (Bharat Serums and Vaccines) polyvalent antivenom	58	12 (20.7%)	1 vial: 7 (77.8%) 2 vials: 2 (22.2%) ≥3 vials: 0 (0%)	Any reaction: 2 (16.7%) Urticarial rash: 2 (16.7%) Fever: 2 (16.7%) Bronchospasm: 0 (0%) Hypotension: 0 (0%)
2017–2018	Snake Venom Antiserum African (VINS Bioproducts) polyvalent antivenom	44	9 (20.5%)	1 vial: 4 (44.4%) 2 vials: 4 (44.4%) ≥3 vials: 1 (11.1%)	Any reaction: 1 (11.1%) Urticarial rash: 1 (11.1%) Fever: 0 (0%) Bronchospasm: 0 (0%) Hypotension: 0 (0%)
2019–2021	Inoserp Pan-Africa (Inosan Biopharma) polyvalent antivenom	83	25 (30.1%)	1 vial: 9 (42.9%) 2 vials: 8 (38.1%) ≥3 vials: 4 (19.0%)	Any reaction: 5 (20%) Urticarial rash: 4 (16%) Fever: 1 (4%) Bronchospasm: 0 (0%) Hypotension: 1 (4%)

Since 2016, intermittent stocks of SAIMR (South African Vaccine Producers) polyvalent antivenom have been available through charitable donation by the Bio-Ken Snake Farm in Watamu.

**Fig 6:**
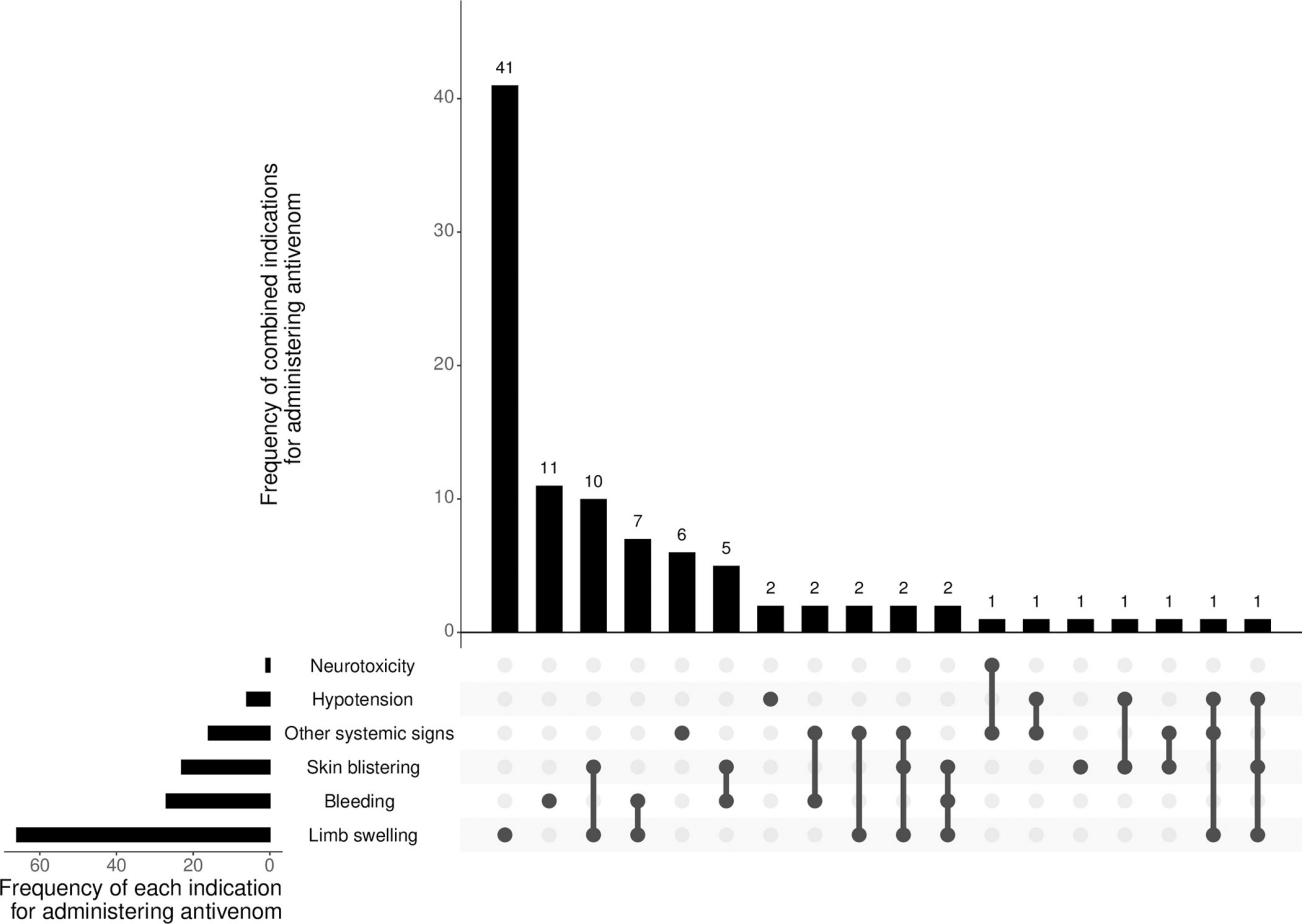
Upset plot of indications for administering antivenom to children with snakebite at Kilifi County Hospital. Upper bar chart, x axis: list of the various combinations of indications for administering antivenom; y axis: number of children that had each combination of antivenom indications. Lower left bar chart, x axis: total numbers of children that had each indication for antivenom; y axis: list of individual indications for administering antivenom. Other systemic signs included excess salivation, vomiting, and restlessness.

Antivenom was administered for limb swelling in 66 (55.5%) cases, and swelling was the sole indication in 41 (34.5%) cases (Fig 6). Other indications for antivenom included: bleeding in 27 (22.7%) cases, skin blistering in 23 (19.3%), hypotension in 6 (5.0%), restlessness in 12 (10.1%), vomiting in 6 (5.0%), excess salivation in 5 (4.2%), and neurotoxicity in 1 (0.8%) case (in a 2^nd^ case of neurotoxicity, data were missing on whether antivenom was administered).

An acute allergic reaction to antivenom occurred in 21 (17.6%) cases (Table 2). A rash occurred in 19 (16.0%) cases, fever in 4 (3.4%), anaphylactic bronchospasm in one (0.8%), and anaphylactic hypotension in one (0.8%) case. The case with anaphylactic hypotension subsequently died due to this complication (Table 3). Amongst children receiving antivenom, 7 (5.9%) received subcutaneous adrenaline (as treatment, rather than prophylaxis), 19 (16.0%) received intravenous hydrocortisone, and 20 (16.8%) received chlorphenamine (Table C in S1 Text).

**Table 3:** Characteristics of snakebite associated paediatric deaths.

	Age category (years)	Year of bite	Location of death	Time from bite until attended hospital (hours)	Time from admission to death (days)	Anatomical site of the snakebite	Circumstances of the snakebite	Traditional therapies received	Vital signs on admission (pulse rate, respiratory rate, O_2_ saturations, systolic blood pressure, and axillary temperature)	Received antivenom	Developed severe local tissue damage	Cause of death (hospital records and verbal autopsy)
1	0–5	2007	KCH	5	0	Left leg	Inside home	None reported	139 beats/min; 24 breaths/min; 97%; NA; 35.3°C	No	No	Snakebite
	**Features of envenoming:** Bite site swelling, hypotension, bradycardia **Clinical narrative:** Presented in a critical condition with hypotensive shock, bradycardia, and respiratory distress. Managed with cardiopulmonary resuscitation, adrenaline, and atropine, but died shortly after admission.											
2	0–5	2012	KCH	24	1	Right leg	NA	Puncture to bite site	181 beats/min; 58 breaths/min; 99%; 123 mmHg; 37.8°C	One vial	No	Snakebite
	**Features of envenoming:** Bite site swelling **Clinical narrative:** Sustained a snakebite to the toe. Delayed presentation by 1-day as sought cutting to bite site by a traditional healer. On presentation was irritable and unable to eat. Became progressively lethargic and died following a cardiac arrest the day following admission.											
3	6–12	2019	KCH	NA	0	NA	NA	NA	66 beats/min; 20 breaths/min; 89%; NA; 39.0°C	NA	NA	Snakebite
	**Features of envenoming:** Neurotoxicity **Clinical narrative:** Bitten by a long brown snake and within hours developed agitation, excessive salivation, difficulty in breathing, and confusion. Initially presented to a local dispensary and then referred to Kilifi County Hospital. Lost consciousness prior to admission and died on the day of admission.											
4	6–12	2020	KCH	12	1	Right foot	While walking on a footpath	None reported	122 beats/min; 20 breaths/min; 100%; 112 mmHg; 33.9°C	Two vials	No	Snakebite
	**Features of envenoming:** Bite site swelling, and neurotoxicity **Clinical narrative:** Snakebite to the lower limb was followed by difficulty in swallowing and blurred vision. At hospital developed respiratory arrest which was managed with mechanical ventilation. The following day spontaneous breathing returned, and mechanical ventilation was ceased. Subsequently developed copious haematemesis followed by cardiac arrest. Managed with cardiopulmonary resuscitation and adrenaline but died shortly after.											
5	6–12	2020	KCH	NA	0	Unspecified lower limb	Farming	NA	116 beats/min; 24 breaths/min; 100%; NA; 33.7°C	NA	NA	Antivenom associated anaphylaxis
	**Features of envenoming:** Bite site swelling **Clinical narrative:** Initially presented to a dispensary following snakebite to the lower limb and was then transferred to Kilifi County Hospital. At admission there was severe swelling up to mid-thigh, bleeding from the bite site, agitation, and confusion. Following two doses of antivenom, developed new onset hypotension, tachypnoea, chest pain, and dizziness. Managed as anaphylaxis and adrenaline was administered. Condition progressed to cardiopulmonary arrest which did not respond to further doses of adrenaline and manual ventilation.											

KCH: Kilifi County Hospital; mmHg: millimetres of mercury; NA: missing data or unrecordable parameter.

Antimicrobials were administered to 299 (63.3%) children with snakebite. The most frequently used antimicrobials were cloxacillin (263 cases; 55.7%), gentamicin (110 cases; 23.3%), and metronidazole (57 cases; 12.1%) (Table C in S1 Text).

The mean duration of hospital stay was 6.3 days (SD 17.8 days). There was an average of 195 bed-days occupied per year due to paediatric snakebite admissions. The mean duration of hospital stay was significantly prolonged (55.0 days vs 4.2 days; p<0.001) in cases with severe local tissue damage (defined as developing skin necrosis or undergoing local surgery). Amongst the 25 cases with severe local tissue damage, 20 (80.0%) were admitted for ≥7-days.

### Predictors of severe local tissue damage

Severe local tissue damage developed in 25 cases (4.3%). Necrosis at the site of the bite developed in 22 cases (3.8%), and 19 (3.3%) required surgery. Ten cases underwent debridement, seven had a fasciotomy, four underwent skin grafting, and three had an amputation. The three cases fulfilling the criteria for severe local tissue damage that did not have skin necrosis had all undergone fasciotomy. All the cases that underwent amputation (n = 3) were preceded by the development of skin necrosis, and none were preceded by surgical fasciotomy.

Following multiple imputation, the following covariates were assessed in a univariate logistic regression analysis to identify potential predictors of severe local tissue damage: age, MUAC-for-age z-score, site of bite (upper vs lower limb), time from bite to admission, vital signs on admission (axillary temperature, pulse rate, respiratory rate, systolic blood pressure, capillary refill time in seconds, and oxygen saturations), serum sodium, serum potassium, eGFR, white blood cell count, granulocyte count, lymphocyte count, platelet count, and haemoglobin on admission. In the univariate analysis the following covariates were identified with p≤0.10: upper limb bite (OR 3.38; 95% CI 1.34–8.56; p = 0.01); white blood cell count (OR 1.17; 95% CI 1.10–1.24; p<0.01); granulocyte count (OR 1.16; 95% CI 1.08–1.24; p<0.01); lymphocyte count (OR 1.14; 95% CI 1.00–1.30; p = 0.06); systolic blood pressure (OR 1.03; 95% CI 1.00–1.06; p = 0.04), pulse rate (OR 1.02; 95% CI 1.00–1.03; p = 0.03); serum sodium (OR 0.88; 95% CI 0.81–0.97; p = 0.01); oxygen saturations (OR 0.85; 95% CI 0.72–1.01; p = 0.06); and haemoglobin (OR 0.69; 95% CI 0.57–0.84; p<0.01) (Table 4).

**Table 4:** Univariate and multivariate logistic regression analysis of predictors of severe local tissue damage.

Covariate	All cases	None-severe local tissue damage	Severe local tissue damage	Missing, n (%)	Univariate analysis		Multivariate analysis	
					OR (95% CI)	p-value	OR (95% CI)	p-value
**Age, mean (SD)**	7.6 (3.1)	7.6 (3.1)	7.6 (3)	0 (0)	1 (0.88–1.14)	1	-	-
**MUAC-for-age z-score, mean (SD)**	-1.2 (1.1)	-1.2 (1.1)	-1.2 (1.1)	25 (4.3)	1.06 (0.71–1.58)	0.77	-	-
**Upper limb (%)**	14.3 (-)	13.4 (-)	34.7 (-)	174 (29.8)	3.38 (1.34–8.56)	0.01	3.27 (1.17–9.17)	0.03
**>12-hour delay bite to admission (%)**	31 (-)	30.9 (-)	33.3 (-)	167 (28.6)	1.12 (0.46–2.74)	0.8	-	-
**Pulse rate, mean (SD)**	116.2 (23.5)	115.8 (23.3)	126.3 (27.4)	1 (0.2)	1.02 (1–1.03)	0.03	1 (0.98–1.02)	0.95
**Respiratory rate, mean (SD)**	28.3 (6.7)	28.2 (6.7)	29.9 (5.8)	2 (0.3)	1.03 (0.98–1.09)	0.22	-	-
**Systolic blood pressure, mean (SD)**	112.7 (15)	112.4 (14.9)	119.4 (15.3)	129 (22.1)	1.03 (1–1.06)	0.04	1.03 (1–1.07)	0.04
**Capillary refill time >2 seconds (%)**	1.6 (-)	1.5 (-)	5.3 (-)	7 (1.2)	1.27 (0.77–2.09)	0.34	-	-
**Axillary temperature, mean (SD)**	36.8 (0.7)	36.8 (0.7)	36.8 (0.9)	1 (0.2)	0.89 (0.52–1.53)	0.68	-	-
**Oxygen saturation, mean (SD)**	98.9 (1.6)	99 (1.5)	98.3 (3)	2 (0.3)	0.85 (0.72–1.01)	0.06	1.01 (0.81–1.26)	0.93
**Haemoglobin, mean (SD)**	11.6 (1.6)	11.7 (1.5)	10.4 (2.9)	53 (9.1)	0.69 (0.57–0.84)	0	0.72 (0.56–0.92)	0.01
**WCC, mean (SD)**	11.4 (4.8)	11.2 (4.6)	16.5 (6.5)	56 (9.6)	1.17 (1.1–1.24)	0	1.14 (1.06–1.22)	0
**Granulocyte count, mean (SD)**	8.3 (4.5)	8.1 (4.3)	12.4 (7.3)	138 (23.6)	1.16 (1.08–1.24)	0	-	-
**Lymphocyte count, mean (SD)**	3 (2.1)	3 (2.1)	3.9 (3.3)	76 (13)	1.14 (1–1.3)	0.06	-	-
**Platelet count, mean (SD)**	341.7 (111.9)	342.5 (110.9)	323.6 (130.4)	54 (9.2)	1 (1–1)	0.41	-	-
**Sodium, mean (SD)**	136.5 (4.2)	136.6 (4.1)	133.9 (5.1)	224 (38.4)	0.88 (0.81–0.97)	0.01	0.9 (0.82–0.99)	0.03
**Potassium, mean (SD)**	3.9 (0.6)	3.9 (0.6)	3.8 (0.5)	223 (38.2)	0.74 (0.37–1.48)	0.39	-	-
**eGFR, mean (SD)**	149.6 (50.5)	149.8 (50.1)	146.4 (59.8)	344 (58.9)	1 (0.99–1.01)	0.79	-	-

CI: confidence interval; eGFR: estimated glomerular filtration rate; MUAC: mid-upper arm circumference; OR: odds ratio; SD: standard deviation; WCC: white cell count

Lymphocyte count and granulocyte count were omitted from the multivariate logistic regression model, as each were positively associated with severe local tissue damage and, therefore, the total white blood cell count was selected (to avoid multicollinearity). The rate of severe local tissue damage was similar between bites to the arm and the hand, although the event rate was small. Two participants (13.3%) with arm bites developed severe local tissue damage, five (11.9%) with hand bites, six (4.3%) with leg bites and seven (3.3%) with foot bites (Fig 4). Therefore, the covariate of upper limb bite (hand or arm) was entered into the multivariate analysis. The following statistically significant predictors were identified from the multivariate analysis: upper limb bite site (OR 3.27; 95% CI 1.17–9.17; p = 0.03); white cell count (OR 1.14; 95% CI 1.06–1.22; p<0.01); systolic blood pressure (OR 1.03; 95% CI 1.00–1.07; p = 0.04); serum sodium (OR 0.9; 95% CI 0.82–0.99; p = 0.03); and haemoglobin (OR 0.72; 95% CI 0.56–0.92; p = 0.01).

### Snakebite associated mortality

Nine of the children in this study have died. Four of these deaths were unrelated to the snakebite and occurred years later during separate hospital episodes. The cause of deaths in these cases were epilepsy, accidental fall, acute respiratory infection associated with HIV/AIDS, and seizures secondary to previous bacterial meningitis, and these deaths occurred 13-, 5-, 5-, and 4-years after the snakebite incident, respectively. Of the five snakebite associated deaths, two were due to neurotoxic envenoming, one had cardiovascular instability (hypotension, bradycardia and respiratory distress), one developed antivenom associated anaphylaxis, and one infant had a general deterioration of an uncertain nature, which culminated in cardio-pulmonary arrest (Table 3). The five snakebite associated deaths occurred within one day of the hospital admission.

## Discussion

This study represents one of the most comprehensive analyses of paediatric snakebite in Africa and demonstrates the substantial burden of this disease. The concerning trend of rising cases of paediatric snakebite in Kilifi, in parallel with population growth, underscores the need for strengthened targeted prevention strategies, improved training of healthcare providers, and increased availability of antivenom treatments. There is also an urgent need for similar studies on the epidemiology of paediatric snakebite envenoming to be conducted, particularly in sites with access to established health and demographic surveillance systems (HDSS) in Africa [18]. There was a substantial fall in hospital-attended snakebite incidence in 2021, which may have been caused by the SARS-CoV-2 pandemic altering health seeking behaviour [19].

One quarter of paediatric snakebite cases were given antivenom, which was most frequently indicated for local envenoming. Most recently (years 2019–2022), Inoserp Pan-Africa (Inosan Biopharma) polyvalent antivenom has been used, although, in 2022 it was withdrawn from the Kenyan market after failing a risk-benefit assessment conducted by the World Health Organization [20]. Since 2016, intermittent stocks of SAIMR (South African Vaccine Producers) polyvalent antivenom have been available through charitable donation by the Bio-Ken Snake Farm in Watamu, which tends to be reserved for more severe cases, given its evidence of pre-clinical efficacy [21]. Although the frequency of severe allergic reactions was low, there was one case that died as a direct result of antivenom induced anaphylaxis. Despite local envenoming being the most frequent indication for administering antivenom in much of Africa, its effectiveness for this indication is unproven, particularly if it is given late, and clinical trials are urgently needed [22]. Novel oral small molecule therapeutics may hold promise, particularly if they can be administered in rural clinics and thus reduce the time to treatment [23–25].

Bleeding was the most common sign of systemic envenoming. Despite this, measures of coagulopathy, such as the 20WBCT, were rarely documented in the case files. Early detection of coagulopathy is important to avoid delays in administering antivenom for systemic envenoming. Following this study, routine clinical practice at Kilifi County Hospital now includes measuring the 20WBCT in all cases of snakebite.

It was not possible to describe the predominant biting species in this study. It is believed that the puff adder (*Bitis arietans*), spitting cobras (*Naja* spp.), and burrowing asps (*Atractaspis* spp.) are the predominant medically important species in this region, but the relative contribution of these, and other less medically important species, is unknown. Mambas (*Dendroaspis* spp.) and non-spitting cobras (*Naja haje*, *N*. *subfulva*) are habitual to this region of Kenya. Although there were only two cases of neurotoxic envenoming in this study, both were fatal.

Delayed presentation to hospital was frequent and often prolonged. As most cases resided within the KHDSS study area, which is near to Kilifi County Hospital, it is likely that there is a delay in the decision to attend hospital. It is concerning that a large proportion of children received traditional therapies prior to presenting to hospital, particularly as this was associated with a statistically significant prolongation of the bite to admission time. The most frequently sought traditional therapy was application of a ‘black stone,’ which has been used in many geographic settings despite its lack of efficacy [26].

Most children in this study received antimicrobials. The majority had cloxacillin, although broader spectrum agents such as ceftriaxone and gentamicin were also used. Unlike other animal bites, snakebite rarely results in infection and routine antimicrobial prophylaxis is not recommended [27,28].

Severe local tissue damage developed in 4.4% of cases and was often associated with admissions that were weeks or even months long. Low haemoglobin was associated with severe local tissue damage. The direction of causality is uncertain, and it is feasible that children with anaemia may have other comorbidities that put them at risk of local tissue damage. Snakebite can cause anaemia as a result of thrombotic microangiopathy, although this is usually associated with thrombocytopaenia, which was uncommon in this study [29]. A raised white cell count on admission was also associated with severe local tissue damage, which has been demonstrated in other settings [30,31]. This is likely to be a bi-directional process, with activation of the innate immune system causing collateral damage at the bite site, and damage of local tissues triggering an immune response. Children that had sustained a snakebite to the upper limb were more likely to develop severe local tissue damage, the reason for which is uncertain. It is regarded that children are at a greater risk of envenoming, compared to adults, as they receive a higher dose of venom relative to their body weight; therefore, it may follow that the small upper limb of a child is particularly at risk. Increasing systolic blood pressure and lower serum sodium were associated with severe local tissue damage, although the small effect size and borderline statistical significance make the clinical relevance of these associations uncertain. Ultimately, further studies of local envenoming in sub-Saharan Africa are needed to confirm whether the predictors of severity identified in this single site study are reproducible.

A limitation of this study was that paediatric snakebite cases that did not attend hospital were missed, and therefore the true burden of disease has been under-estimated. A household survey is needed to further define the epidemiology of snakebite in Kilifi. Although the KHDSS study enabled reliable identification of consecutive cases of paediatric snakebite, with routine data collection and clinical laboratory analyses, the KHDSS study was not specifically designed to study snakebite. Thus, many important datapoints, such as whether antivenom was administered, needed to be retrospectively collected from the hospital records. Nevertheless, documentation on the paediatric HDU tended to be detailed and accurate, with standardised admission and discharge case report forms and contemporaneous daily documentation during admission. All cases were managed on a paediatric HDU which is supported and staffed by the KEMRI-Wellcome Trust Research Programme. There were missing data, particularly for biochemistry laboratory results and for items with variable documentation in the clinical records, such as the use of traditional therapies. The risk of bias due to missing data was partially mitigated using multiple imputation.

In conclusion, this study demonstrates the substantial burden of snakebite envenoming amongst children in rural Kenya. This is traumatic for children, interrupts schooling and development, is disruptive for families, places a substantial burden on healthcare facilities, and can lead to permanent disability or death. There is an urgent need for improved community awareness, with particular focus on preventative strategies, appropriate first aid, and the importance of early presentation to hospital. Many children in Kilifi receive antivenom for local envenoming, and it is important to assess whether this is effective.

## Supporting information

S1 Text **Table A. Proportion of paediatric admissions to Kilifi County Hospital that were due to snakebite.** HDU: high dependency unit; KCH: Kilifi County Hospital. During the period between 2003 and 2005 the numbers of snakebite admissions are underestimated as the hospital surveillance system was not yet fully established. **Table B. Clinical laboratory abnormalities on admission in cases of paediatric snakebite attending Kilifi County Hospital.** eGFR: estimated glomerular filtration rate. The KEMRI-Wellcome Trust Research Programme age-adjusted Kilifi paediatric reference ranges were used (version 2.0 September 2021). **Table C**. Administration of antimicrobials and treatments for antivenom associated allergic reactions in cases of paediatric snakebite attending Kilifi County Hospital.
(DOCX)

S1 Data The de-identified final dataset for the study population. (CSV)

S1 Fig Upset plot of traditional therapies sought prior to presenting to hospital amongst children with snakebite at Kilifi County Hospital. Upper bar chart, x axis: list of the various combinations of traditional therapies that were sought prior to attending Kilifi County Hospital; y axis: number of children that had sought each combination of traditional therapies. Lower left bar chart, x axis: total numbers of children that had sought each type of traditional therapy; y axis: list of individual traditional therapies that were sought prior to attending Kilifi County Hospital.
(TIF)
