# Supplementary material for: A long-term observational study of paediatric snakebite in Kilifi County, south-east Kenya
Source: PLoS Negl Trop Dis. 2023 Jul 17;17(7):e0010987. doi: 10.1371/journal.pntd.0010987 (PMC10403087; doi:10.1371/journal.pntd.0010987)
Supplement: S1 Text — Table A. Proportion of paediatric admissions to Kilifi County Hospital that were due to snakebite. HDU: high dependency unit; KCH: Kilifi County Hospital. During the period between 2003 and 2005 the numbers of snakebite admissions are underestimated as the hospital surveillance system was not yet fully established. Table B. Clinical laboratory abnormalities on admission in cases of paediatric snakebite attending Kilifi County Hospital. eGFR: estimated glomerular filtration rate. The KEMRI-Wellcome Trust Research Programme age-adjusted Kilifi paediatric reference ranges were used (version 2.0 September 2021). Table C. Administration of antimicrobials and treatments for antivenom associated allergic reactions in cases of paediatric snakebite attending Kilifi County Hospital. (DOCX) [file pntd.0010987.s001.docx]

**Table A in S1 Text. Proportion of paediatric admissions to Kilifi County Hospital that were due to snakebite**

| **Year** | **Paediatric admissions to KCH, n** | **Paediatric admissions to KCH HDU, n** | **Paediatric snakebite admissions, n** | **Percentage of paediatric admissions to KCH due to snakebite** | **Percentage of paediatric HDU admissions to KCH due to snakebite** |
| --- | --- | --- | --- | --- | --- |
| 2003 | 5,533 | 920 | 11 | 0.20 | 1.20 |
| 2004 | 5,038 | 775 | 15 | 0.30 | 1.94 |
| 2005 | 4,724 | 828 | 18 | 0.38 | 2.17 |
| 2006 | 4,995 | 735 | 34 | 0.68 | 4.63 |
| 2007 | 4,477 | 869 | 35 | 0.78 | 4.03 |
| 2008 | 4,358 | 933 | 24 | 0.55 | 2.57 |
| 2009 | 4,812 | 964 | 36 | 0.75 | 3.73 |
| 2010 | 4,347 | 988 | 34 | 0.78 | 3.44 |
| 2011 | 4,204 | 981 | 37 | 0.88 | 3.77 |
| 2012 | 3,618 | 936 | 20 | 0.55 | 2.14 |
| 2013 | 2,831 | 938 | 27 | 0.95 | 2.88 |
| 2014 | 4,061 | 1,159 | 41 | 1.01 | 3.54 |
| 2015 | 4,233 | 1,204 | 40 | 0.94 | 3.32 |
| 2016 | 3,778 | 1,170 | 42 | 1.11 | 3.59 |
| 2017 | 2,250 | 1,115 | 23 | 1.02 | 2.06 |
| 2018 | 3,923 | 1,261 | 35 | 0.89 | 2.78 |
| 2019 | 4,258 | 1,362 | 58 | 1.36 | 4.26 |
| 2020 | 3,446 | 1,344 | 37 | 1.07 | 2.75 |
| 2021 | 3,152 | 1,124 | 17 | 0.54 | 1.51 |
| **Total:** | **78,038** | **19,606** | **584** | **0.75** | **2.98** |

HDU: high dependency unit; KCH: Kilifi County Hospital.

During the period between 2003 and 2005 the numbers of snakebite admissions are underestimated as the hospital surveillance system was not yet fully established.

**Table B in S1 Text. Clinical laboratory abnormalities on admission in cases of paediatric snakebite attending Kilifi County Hospital**

| **Parameter** | **N (%)** |
| --- | --- |
| Anaemia | 51 (9.6) |
| Leukocytosis | 314 (59.5) |
| Neutrophilia | 259 (63.5) |
| Eosinophilia | 37 (9.1) |
| Basophilia | 145 (35.6) |
| Monocytosis | 146 (35.9) |
| Lymphocytosis | 54 (10.6) |
| Thrombocytosis | 45 (8.5) |
| Thrombocytopaenia | 22 (4.2) |
| Hyperkalaemia | 4 (1.1) |
| Hypokalaemia | 60 (16.6) |
| Hypernatraemia | 11 (3.1) |
| Hyponatraemia | 55 (15.3) |
| eGFR <90 | 12 (5.0) |

eGFR: estimated glomerular filtration rate.

The KEMRI-Wellcome Trust Research Programme age-adjusted Kilifi paediatric reference ranges were used (version 2.0 September 2021).

**Table C in S1 Text. Administration of antimicrobials and treatments for antivenom associated allergic reactions in cases of paediatric snakebite attending Kilifi County Hospital**

| **Antimicrobial treatment:** | **N (%)** |
| --- | --- |
| Any antimicrobial | 299 (63.3) |
| Cloxacillin | 263 (55.7) |
| Gentamicin | 110 (23.3) |
| Metronidazole | 57 (12.1) |
| Ceftriaxone | 19 (4.0) |
| Penicillin | 13 (2.8) |
| Ampicillin | 12 (2.5) |
| Chloramphenicol | 4 (0.8) |
| Other | 9 (1.9) |
| **Treatments for antivenom associated acute allergic reactions:** |  |
| Intravenous chlorphenamine | 20 (16.8) |
| Intravenous hydrocortisone | 19 (16) |
| Subcutaneous adrenalin | 7 (5.9) |
