# Supplementary figures and images for: A long-term observational study of paediatric snakebite in Kilifi County, south-east Kenya
Source: PLoS Negl Trop Dis. 2023 Jul 17;17(7):e0010987. doi: 10.1371/journal.pntd.0010987 (PMC10403087; doi:10.1371/journal.pntd.0010987)

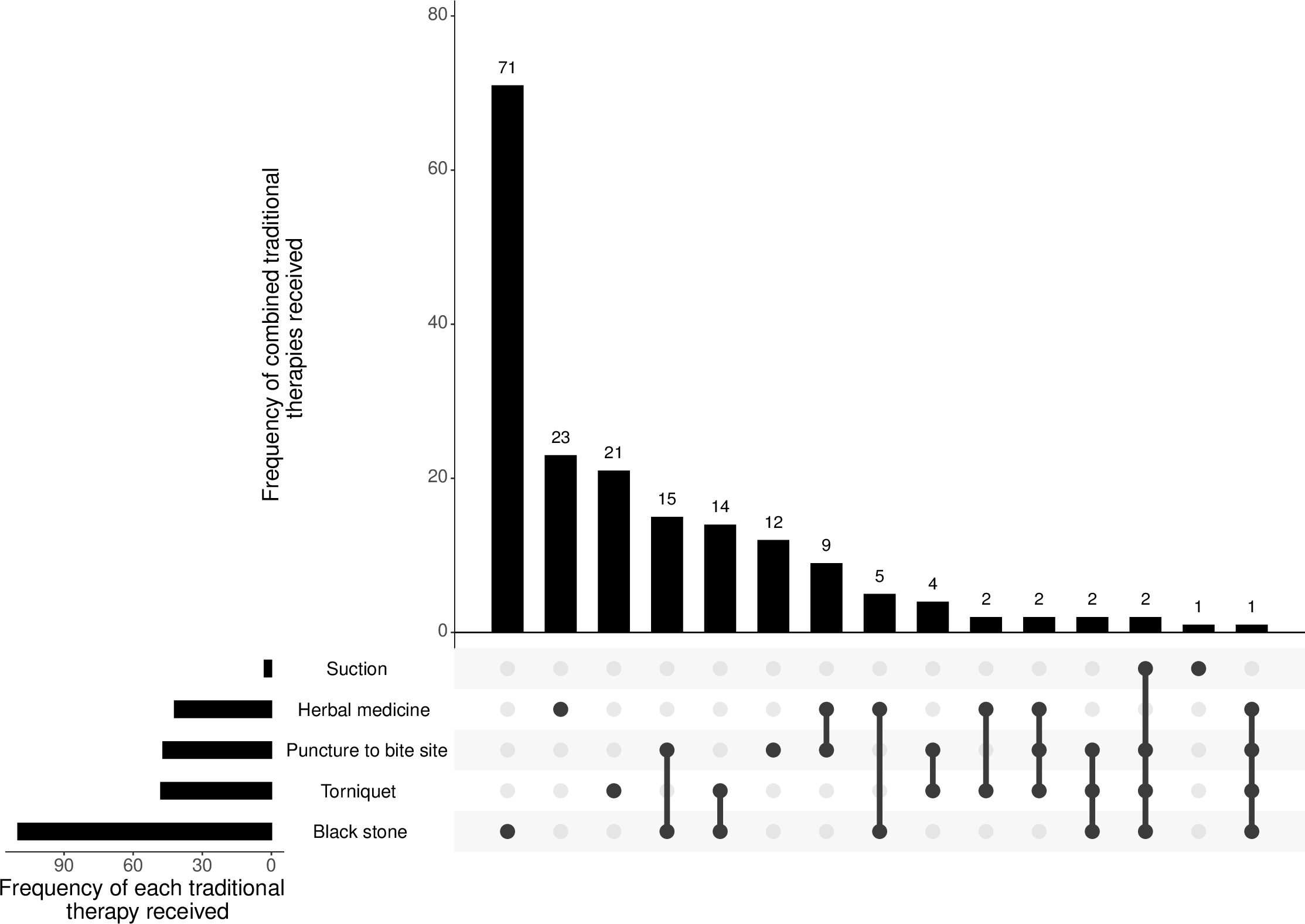

Supplement: S1 Fig — Upper bar chart, x axis: list of the various combinations of traditional therapies that were sought prior to attending Kilifi County Hospital; y axis: number of children that had sought each combination of traditional therapies. Lower left bar chart, x axis: total numbers of children that had sought each type of traditional therapy; y axis: list of individual traditional therapies that were sought prior to attending Kilifi County Hospital. (TIF) [file pntd.0010987.s003.tif]
